# Dr. Buchanan on Lithoplatomy
*Glasgow Journal, No. XII.


**Published:** 1831-01-01

**Authors:** 


### LXVI.

### Dr. Buchanan on Lithoplatomy.*

We may inform our readers at starting, that lithoplatomy signifies dilatation of the urethra for the extraction of a stone. Dr. Buchanan has written a very interesting paper on the subject in our valuable contemporary of Glasgow. The remarks, to which we shall proceed forthwith, were occasioned by two cases which occurred to Dr. B., in one of which three stones were extracted from the female urethra; and in the second, a stone measuring two inches and eight lines in its largest circumference, and one inch and ten lines in its smallest, was extracted from the membranous part of the male urethra by cutting down upon the grooved staff. From the consideration of these cases Dr. Buchanan proceeds to inquire into the ancient and modern methods of extracting calculi from the urethra of the male, namely, curved probes, scoops, forceps of various kinds, *suction by the mouth*, the application of a pump, &c. The Egyptians compressed the neck of the bladder, and inflated the urethra through a wooden canula, and introduced elastic tubes which they distended in the bladder; and then pushed the stone forwards from the neck of the bladder. The latest and the best means of extracting calculi of small size are the forceps invented by Sir Astley Cooper and Mr. Weiss. The instrument for dilating the female urethra by the same ingenious instrument-maker, has superseded all other modes of operating for stone in the female bladder. Dr. Buchanan has objections to it.

"The objections which I have from experience to urge against Mr. Weiss' instrument are the following: 1st, The difficulty of introducing the two blades at once into the bladder, from the increase of their thickness towards the screw or handle. 2d, The unequal manner in which the canal is dilated, the exterior part requiring to yield before the interior. 3d, The liability to laceration of the mucous membrane, in consequence of one part being put more on the stretch than another. 4th, The tendency of the instrument to slip out of the passage, while the dilatation is effecting. 5th, The canal being dilated in a very unfavourable direction by only *two lateral* blades, the rami of the ischia present on each side a natural barrier to their expansion."

The speculum vaginæ of Mr. Weiss, a very excellent instrument, has given Dr. Buchanan the hint for a speculum urethræ, or *litho-platome*, which he has had constructed. It is on precisely the same principle as the speculum vaginæ, but is much less in size, and instead of being hollow it has for strength been made solid. By means of it a full view of the female bladder is readily obtained with a properly directed light. Now the question arises as to the possibility of dilating the male urethra in a similar manner. Dr. Buchanan feels convinced that stones of nearly as large a size might spontaneously pass by the male as by the female urethra. Of this we entertain considerable doubts. That calculi of some size can be brought from the bladder to the membranous part of the urethra we well know, but beyond this their extraction becomes more difficult. In short, we do not consider it probable that calculi beyond a certain size will be withdrawn through the urethra by dilatation, or at least

* Glasgow Journal, No. XII.

that such an operation will come into general use. We rather imagine that the chief modern improvement in this part of surgery will proceed from the removal of calculi from the bladder, when *small*, by the urethral forceps, and by our better acquaintance with the conditions of the urine, the states of system that they indicate and by which they are occasioned, and the most philosophical methods of treating those conditions of the urinary secretion. But to proceed. If objections should be urged against extreme dilatation of the urethra, Dr. Buchanan has another string to his bow, twice as long and strong as that.

"The operation I allude to, is that by which the male urethra is converted into a female one, and thus the difficulty and danger of both lithotomy and lithotrity is at one step superseded by lithoplatomy.

For the purpose of effecting the first part of this operation, the patient must be secured as for lithotomy; a grooved staff is introduced into the bladder; the membranous part of the urethra near the prostate incised, to the necessary extent, to admit the lithoplatome; and then, having withdrawn the staff, the operation of dilatation of the remaining part of the urethra, prostate, and neck of the bladder, will be, with nearly the same ease, safety and effect, accomplished, by means of my lithoplatome, as in the female.

That there are many objections to the above method of operating for stone I must at once admit, and until I can give it a fair trial on the living subject, I shall not contrast it either with lithotomy or lithotrity; judging, however, from what I have already experienced in the female urethra, and, on the dead subject, in the male, I am not the least afraid of the result. I shall only add further, that if the operation of lithoplatomy does come up to my expectations, and that of many judicious and talented surgeons here, to whom I have mentioned it, how much will suffering humanity be relieved, and the labours of the surgeon facilitated."

Now it is a singular fact that this idea occurred to Mr. Keate some few years ago, and that he made experiments on the dead body in St. George's Hospital, with the view of determining the advantages or otherwise of the proceeding. He was induced, however, to refrain from performing the operation in the living body on two accounts; first, because a moderately sized stone can be extracted from a healthy bladder with very little risk: and secondly, because a large stone could not safely be extracted from an unhealthy bladder, even if it could be extracted at all, by this dilatation of the membranous part of the urethra.

We shall make no further comments on Dr. Buchanan's proposal. That the object of the Doctor is most laudable we are sure, and we cordially award him the meed of perseverance and of research in the prosecution of his experiments and inquiries. We fear, however, that we cannot quite participate in the excusable enthusiasm with which he anticipates the most brilliant results from this operation.

